# Adipogenic Signaling Promotes Arrhythmia Substrates before Structural Abnormalities in TMEM43 ARVC

**DOI:** 10.3390/jpm12101680

**Published:** 2022-10-09

**Authors:** Sunil K. Vasireddi, Prasongchai Sattayaprasert, Dandan Yang, Adrienne T. Dennis, Emre Bektik, Ji-dong Fu, Judith A. Mackall, Kenneth R. Laurita

**Affiliations:** 1 Heart and Vascular Research Center, MetroHealth Campus, Case Western Reserve University, Cleveland, OH 44106, USA; 2 Stanford Cardiovascular Institute, Department of Medicine, Stanford University, Palo Alto, CA 94305, USA; 3 Deaconess Health System, Evansville, IN 47710, USA; 4 The Dorothy M. Davis Heart and Lung Research Institute, Frick Center for Heart Failure and Arrhythmia, Department of Physiology and Cell Biology, The Ohio State University, Columbus, OH 43210, USA; 5 Cardiovascular Division, Department of Medicine, Brigham and Women’s Hospital, Harvard Medical School, Boston, MA 02115, USA; 6 Harrington Heart and Vascular Institute, University Hospitals Cleveland Medical Center, Cleveland, OH 44106, USA

**Keywords:** arrhythmogenic right ventricular cardiomyopathy, TMEM43, adipogenic, mesenchymal stem cell, IGF-1, arrhythmia, action potential, optical mapping, induced pluripotent stem cells, fibro-fatty infiltrate

## Abstract

Arrhythmogenic right ventricular cardiomyopathy (ARVC) is a genetic disorder of desmosomal and structural proteins that is characterized by fibro-fatty infiltrate in the ventricles and fatal arrhythmia that can occur early before significant structural abnormalities. Most ARVC mutations interfere with β-catenin–dependent transcription that enhances adipogenesis; however, the mechanistic pathway to arrhythmogenesis is not clear. We hypothesized that adipogenic conditions play an important role in the formation of arrhythmia substrates in ARVC. Cardiac myocyte monolayers co-cultured for 2–4 days with mesenchymal stem cells (MSC) were derived from human-induced pluripotent stem cells with the ARVC5 TMEM43 p.Ser358Leu mutation. The TMEM43 mutation in myocyte co-cultures alone had no significant effect on impulse conduction velocity (CV) or APD. In contrast, when co-cultures were exposed to pro-adipogenic factors for 2–4 days, CV and APD were significantly reduced compared to controls by 49% and 31%, respectively without evidence of adipogenesis. Additionally, these arrhythmia substrates coincided with a significant reduction in IGF-1 expression in MSCs and were mitigated by IGF-1 treatment. These findings suggest that the onset of enhanced adipogenic signaling may be a mechanism of early arrhythmogenesis, which could lead to personalized treatment for arrhythmias associated with TMEM43 and other ARVC mutations.

## 1. Introduction

Arrhythmogenic right ventricular cardiomyopathy (ARVC) is a genetic disorder of desmosomal and structural proteins that is characterized by fibro-fatty infiltrate in the ventricles and, importantly, sudden cardiac death (SCD) due to arrhythmia. ARVC accounts for 10–20% of SCD in healthy young patients [1] and there is no cure. Fatal arrythmias in ARVC may precede gross structural changes, which is described as an early concealed phase, and occur in affected patients in their 20–30 s well after cardiac development [2], often during exercise [3,4,5,6]. Patients with ARVC5 due to a TMEM43 mutation, have an exceptionally high risk of sudden death [7]. Crucially, the underlying mechanisms of arrhythmia associated with TMEM43 and, especially, the early concealed phase are not well understood.

Like desmosome mutations associated with ARVC, TMEM43 mutations interfere with β-catenin–dependent transcription through GSK3β activation [8], which enhances adipogenesis by PPAR signaling [9]. Disturbances in PPAR signaling have been associated with ARVC [10], and several studies utilizing ARVC induced pluripotent stem cell (iPSC) derived myocytes have employed pro-adipogenic factors, including PPAR agonists, to coax lipogenesis and calcium dysregulation in myocytes [11,12]. In addition to pro-adipogenic factors, pre-adipocyte/fibroblast non-myocytes in the heart may also be playing an important role in fibro-fatty infiltration [13]. Specifically, the mesenchymal stem cell (MSC) has a broad secretion profile [14] that remodels in the diseased human heart as we [15] and others [16] have shown. Additionally, MSCs have been previously shown to be an important source of fibro-fatty infiltrate in ARVC [17,18]. Despite this, it is unclear if adipogenic conditions, including pro-adipogenic factors and precursor cells, play a role in concealed arrhythmogenesis associated with ARVC.

We hypothesized that adipogenic conditions can play an important role in early, concealed arrhythmogenesis associated with TMEM43 patients. To test this hypothesis, we utilized cardiac myocyte and MSC co-cultures derived from patient-specific iPSCs with the ARVC5 TMEM43 p.Ser358Leu (S358L) mutation that were exposed to pro-adipogenic factors. Optical mapping techniques in mature fiber-aligned co-culture monolayers paced with infrared laser light were used to determine arrhythmia substrates based on abnormal action potential activity. The results of this study could lead to personalized treatment for arrhythmias associated with TMEM43 and other ARVC mutations in general.

## 2. Material and Methods

### 2.1. Differentiation of Human Myocytes from iPSCs

Human iPSC line(s) were kindly provided by Joseph C. Wu, MD, PhD at the Stanford Cardiovascular Institute funded by NHLBI BhiPSC-CVD 75N9202D00019. We differentiated cardiac myocytes from iPSCs with and without the ARVC5 TMEM43 p.Ser358Leu (S358L) mutation using the established protocols by Lian et al. [19] and Tohayama et al. [20]. Additionally, human cardiac myocytes derived from iPSCs (iCells, Cellular Dynamics Inc., Madison, WI, USA) were used as secondary controls. Human iPSC-differentiated myocytes were cultured on plates coated with fibronectin and maintained with media containing RPMI/B27-with insulin. For iCells, plating and maintenance media recommended by the manufacturer were used. For all myocytes, maintenance media was exchanged every 2 days until day 14–21 when experiments were performed.

### 2.2. Differentiation of MSCs from Human iPSCs

MSC differentiation from wildtype human iPSCs or the S358L mutation iPSCs was performed by the continuous repetitive culturing in MSC media for 8–10 passages. MSC culture media was composed of minimum essential medium eagle–alpha modified (α-MEM) with full additives consisting of 10% fetal calf serum (FCS), penicillin/streptomycin, sodium pyruvate, l-ascorbate-2-phosphate, l-Glutamine, non-essential amino acids, and HEPES. Plating was initially in gelatin coated dishes but then transferred to plastic flasks per the protocol outlined by Hynes et al. [21]. All MSCs were used between passage 8–20 consistent with reported use with this method of differentiation. As a positive control, human bone marrow (BM) MSCs were isolated and tested for tri-lineage differentiation potential as previously described [15]. 

### 2.3. TMEM43 Expression Analysis by Quantitative Real-Time RT-PCR (qPCR)

Total RNA was extracted from cell lysis using the TRIzol™ LS Reagent (Invitrogen, Waltham, MA, USA) according to the manufacturer’s instructions and then was used for cDNA synthesis with High-Capacity cDNA Reverse Transcription Kit (Applied Biosystems, Waltham, MA, USA). qRT-PCR analysis of *TMEM43* mRNA level was performed using the SsoFast EvaGreen Supermix with Low Rox (Bio-Rad, Hercules, CA, USA). Gene primers used in the present study: *TMEM43*: forward-ATGGCCGCGAATTATTCCAGT, reverse-GGAGACACCACAAGCGAGA; *GAPDH*: forward-CCAGCAAGAGCACAAGAGGA, reverse-GAGATTCAGTGTGGTGGGGG.

### 2.4. DNA Extraction and TMEM43 Genotyping

Genomic DNA was extracted from the wildtype and S358L human iPSCs, as well as iPSC differentiated cardiomyocytes. The human *TMEM43* sequence was in accordance with NCBI Reference Sequence (http://www.ncbi.nlm.nih.gov/genome, accessed 6 August 2018), and NM_024334.3(TMEM43):c.1073C>T (p.Ser358Leu) was investigated in the present study. The genomic region containing the mutation was amplified using polymerase chain reaction (PCR) with a forward primer (5ʹ- GCGATGACCCTGACCTGGGCCCA-3ʹ) and a reverse primer (5ʹ- TGGCTGGCACCCGTGTCCGAGC-3ʹ). The PCR product was verified by agarose gel electrophoresis and purified for sequencing (Eurofins Genomics, Louisville, KY, USA). The genotype was defined as wild-type (C/C) and heterozygous mutation (C/T).

### 2.5. TMEM43 Total Protein Analysis

Bone marrow, control, and S358L MSCs were harvested from confluent T75 flasks, and total protein (4.53 µg/µL, 4.11 µg/µL, 3.63 µg/µL, respectively) was extracted in 1% Triton Lysis Buffer (1% Triton, 150 mM NaCl, 50 mM Tris pH7.5, 1 mM EDTA) containing Pierce Protease Inhibitor (ThermoScientific, Waltham, MA, USA, cat# A32953). After incubating lysates on ice for 30 min with occasional vortexing, the lysates were centrifuged in a F20 rotor at 18,000 rpm for 30 min at 4 °C. The supernatant lysate was removed, and the protein concentration was determined using the Pierce BCA Protein Assay kit (ThermoScientific, cat# 23227). Samples containing equal amounts of protein were separated by sodium dodecyl sulfate polyacrylamide gel electrophoresis using 10% Mini-Protein TGX precast gels (BioRad cat# 4561035) and then transferred to a PVDF membrane. The membrane was blocked with LI-COR Intercept Blocking Buffer (TBS) for 1 h at room temperature (RT). Primary antibody dilutions were prepared in LI-COR Blocking Buffer (TBS) with 0.2% Tween 20 and incubated overnight at 4 °C. The final antibody concentrations used were 1:5000 of rabbit anti-TMEM43 mAb (Abcam cat# sc-ab184164) and 1:5000 of mouse anti-GAPDH mAb (Sigma Aldrich, St. Louis, MO, USA, cat# G8795-25UL). Membranes were washed 4X (5 min each) with TBS containing 0.1% Tween-20 (TBS-T) at R/T on a bench top shaker. Secondary antibodies conjugated to IRDye 800CW (Goat anti-Rabbit, LI-COR cat# 926-32211) and IRDye 680RD (Goat anti-Mouse, LI-COR cat# 926-68070) were diluted to 1:15,000 in LI-COR Blocking Buffer (TBS) containing 0.2% Tween-20 and 0.01% SDS. Following incubation with appropriate secondary antibodies, the immunoreactive bands were visualized using a LI-COR Odyssey infra-red scanner using Image Studio Version 5.2 imaging software.

### 2.6. Early Adipogenic Condition

The early concealed arrhythmia phase associated with ARVC was simulated by early adipogenic conditions which included co-culturing human cardiac myocytes with human MSCs (precursor adipocytes) and pro-adipogenic factors as follows. All cardiac myocytes were plated at a density of 100,000 cells per well on 3D nanofiber aligned 96–well plates to study mature anisotropic conduction. MSCs (passage 3–5) were plated onto the myocytes at a density of 20,000 cells (1:5 MSC:CM) per well and maintained in media for 2–4 days prior to optical mapping experiments. The benefits of this co-culture assay are that all human cells can be utilized and the independent effects of myocytes, precursor adipocytes (MSCs), and pro-adipogenic factors can be isolated. It is important that MSCs were co-cultured on top of myocytes because it has been shown previously that when mixed prior to plating they can slow impulse propagation. Moreover, we were mostly interested in the paracrine action of MSCs, which we have previously shown to dominate by utilizing transwell inserts and conditioned media [22]. In addition to MSCs, myocyte monolayers were also exposed for 2–4 days at 50–100% of media containing insulin, dexamethasone, IBMX, and the PPAR-gamma agonist rosiglitazone, as used previously to express clinical phenotypes [11]. These adipogenic conditions, which include precursor adipocytes (MSCs) and pro-adipogenic factors, were chosen to represent the early concealed phase when no gross structural abnormalities were observed. After only 3–4 weeks of exposure to pro-adipogenic factors do MSCs differentiate into adipocytes and fat vacuoles appear in myocytes. 

### 2.7. ELISA and Surface Marker Expression

IGF-1 secretion was quantified using Human ELISA kits (R & D Systems Inc., Minneapolis, MN, USA) per standard protocol outlined. Fully confluent monolayers of BM MSCs or iPSC-differentiated MSCs were cultured in 24-well plate with 1 mL culture media for 4 days without media change and the supernatant was used for the ELISA measurements, as we described previously [15]. MSCs used in this study were characterized by well-established surface marker expression methods, as we [15] and others [16] have described previously.

### 2.8. Optical Mapping and Action Potential Analysis

**Figure 1:**
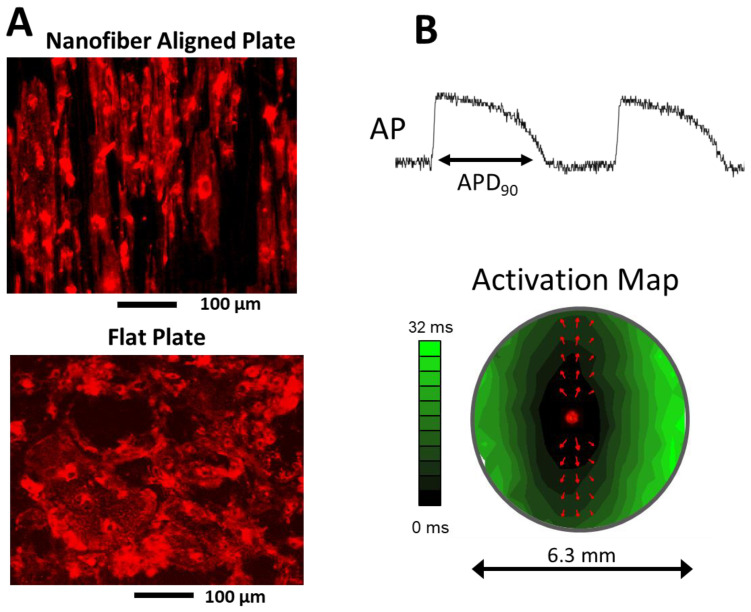
Cardiac myocytes derived from iPSCs and iCells were plated on 3D nanofiber-aligned 96-well plates to obtain a more mature structure (**A**, **top**) compared to a flat plate (**A**, **bottom**). In these examples, cells stained with Di-4-ANEPPS, were plated at a low density so that cell boundaries could be observed. Representative example of an action potential recorded from a single site within a well is shown with the APD_90_ measurement superimposed (**B**, **top**). Activation times determined at each site within a well were used to construct an activation map (**B**, **bottom**) from which local conduction velocity vectors in the longitudinal direction were averaged for each recording (red arrows).

Monolayers were cultured on 3D nanofiber-aligned 96-well plates to obtain a more mature structure (Figure 1A, top) compared to standard flat 96-well plates (Figure 1A, bottom). Monolayers were stimulated with an infrared (IR) laser (1464 nm diode laser) at a cycle length of 0.5 Hz during which simultaneous multi-site (280) action potentials were recorded at room temperature using the voltage sensitive dye Fluovolt (Sigma) as described previously [23]. High-resolution maps of local conduction velocity (CV) vectors at each recording site were determined, from which mean CV in the direction longitudinal to fibers (Figure 1B, red arrows) was determined. Action potential duration (APD) at 90% repolarization (Figure 1B, top, APD_90_) was also determined at each site, and the average across all sites was reported.

## 3. Results

**Figure 2:**
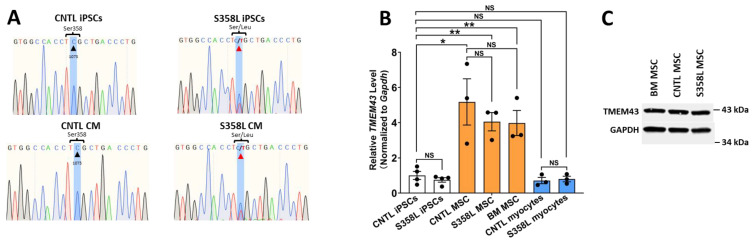
(**Panel A**) shows the DNA-sequencing results for control (CNTL) human iPSCs and patient S358L iPSCs and differentiated CNTL cardiomyocytes (CM) and S358L CM. (**Panel B**) shows the mRNA expression of TMEM43 in CNTL and S358L iPSCs (*n* = 4) and differentiated MSC (*n* = 3) and CM (*n* = 3). (**Panel C**) shows that TMEM43 protein was highly expressed in bone marrow (BM) MSC, CNTL and S358L iPSC differentiated MSCs. (* p < 0.05; ** p < 0.01; ns, nonsignificant).

Critical to our aims, we differentiated cardiac myocytes and MSCs from human wildtype (CNTL) iPSCs and human iPSCs with the S358L mutation. Firstly, we sequenced the genomic DNA of undifferentiated human iPSCs and differentiated myocytes and confirmed the S358L mutation in the patient S358L iPSCs and S358L IPSCs-derived cardiomyocytes (S358L CM, Figure 2A). Secondly, we observed no significant difference in TMEM43 mRNA expression between CNTL and S358L iPSCs, MSCs, and CM (Figure 2B). Finally, high TMEM43 message expressed in all MSCs was consistent with TMEM43 protein expression in all MSCs (Figure 2C). These results validated the TMEM43 S358L mutation in patient iPSCs and their derivatives, and they showed that MSCs highly express TMEM43 at the mRNA and protein levels. 

**Figure 3:**
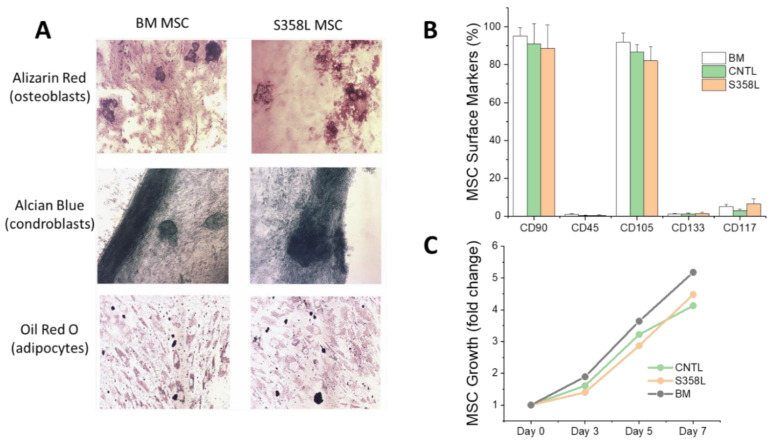
(**A**) shows the multi-lineage potential of bone marrow (BM) MSCs (as a positive control) and MSCs derived from S358L iPSCs. Both BM and S358L MSCs are shown to have a similar potential for differentiation into to bone (stained with Alizarin Red), cartilage (stained with Alcian Blue) and fat (stained with Oil Red O). (**B**,**C**) show that BM MSCs and MSCs derived from S358L iPSCs and control (CNTL) iPSCs have a similar cell surface marker expression and growth rate, respectively (*n* = 3 for all).

To confirm successful differentiation of MSCs from IPSCs, multi-lineage potential (gold standard for MSCs) was performed for S358L MSCs and human BM MSCs (positive control). As shown in Figure 3A, BM and S358L MSCs exhibited a similar potential to differentiate into osteoblasts, chondroblasts, and adipocytes. We also characterized cell-surface markers (Figure 3B) and found that all BM, CNTL, and S358L MSCs were CD90+, CD105+, and CD45-, which are the primary characteristic of MSCs. All three MSC types also exhibited low expression of CD 117 and CD 133 (markers for hematopoietic cells including endothelial cells). Finally, all MSC growth rates (Figure 3C) and doubling times (data not shown) were similar. These results demonstrate that, by using standard protocols, both CNTL and S358L iPSCs could be efficiently differentiated into MSCs and that the cellular composition contains a significant and similar number of MSCs, with no leukocytes (CD45+) or endothelial cells. To the best of our knowledge, this is the first time ARVC iPSC derived MSCs have been studied.

**Figure 4:**
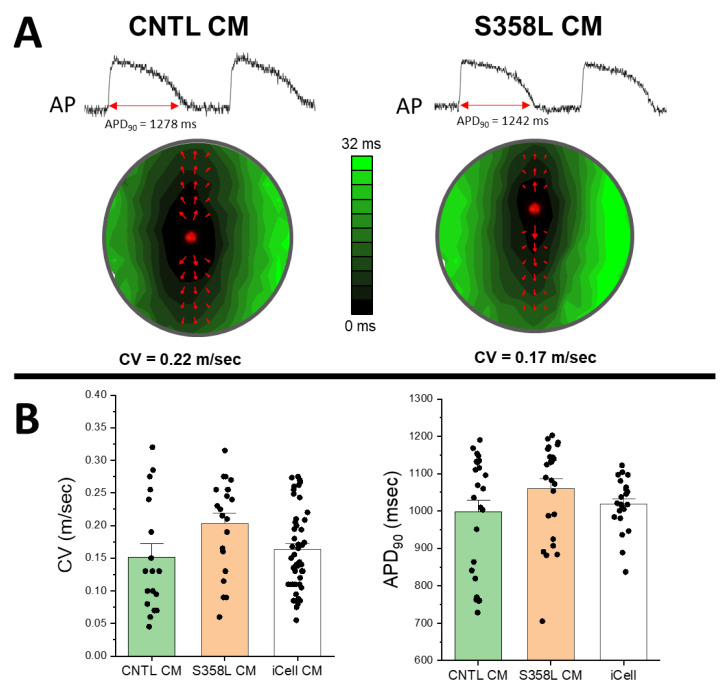
Representative examples of action potentials and activation maps (**A**) recorded during IR pacing at 2000 ms cycle length from wells with cardiac myocytes (CM) alone that were derived from control (left, CNTL) and S358L (**right**) iPSCs. Action potentials and activation maps show a similar APD_90_, activation pattern, and conduction velocity (red arrows) for CNTL CM and S358L CM. Summary data (**B**) show CV (**left**) and APD_90_ (right) measured for each well over multiple differentiations (for iPSC derived CM) and vials (iCells). No significant differences were observed for CV and APD_90_ across all groups. For CV: CNTL CM, *n* = 18; S358L CM, *n* = 21, iCell CM *n* = 47. For APD_90_: CNTL CM *n* = 25; S358L CM, *n* = 25; iCell CM, *n* = 22.

Having confirmed the genome, RNA message, TMEM43 protein expression, and MSC differentiation, we then determined the electrophysiological phenotype of the TMEM43 S358L mutation in cardiac myocytes alone. In Figure 4A are representative examples of action potential traces and activation maps depicting impulse propagation in control (CNTL) and S358L monolayers. In these examples, the S358L mutation did not significantly change action potential morphology, APD, activation pattern, or CV. Since it is well known that iPSC derived myocytes exhibit significant variability, results were pooled across multiple differentiations. Figure 4B shows that when pooled, CV and APD were not significantly different for CNTL and S358L myocytes. Additionally, CV and APD measured in iCell monolayers pooled from multiple vials/lots were also not significantly different compared to CNTL and S358L myocytes. These findings suggest that the TMEM43 S358L mutation in myocytes alone cannot explain the early clinical arrhythmia phenotype associated with TMEM43.

**Figure 5:**
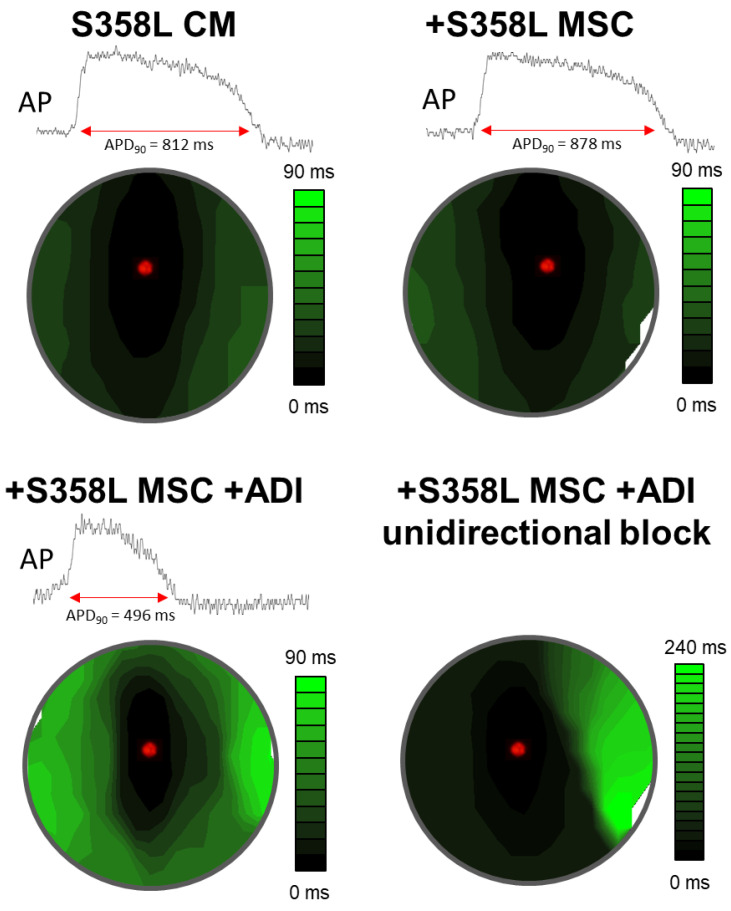
Representative action potential traces and activation maps for S358L cardiac myocytes (CM) alone (**top left**), or co-cultured with 3358L MSCs (+S358L MSC, **top right**), or co-cultured with S358L MSCs plus pro-adipogenic factors (+S358L MSC +ADI, bottom). Only with pro-adipogenic factors was shorter APD_90_ and slower CV (crowding of isochrone lines) observed (**bottom left**). Additionally, in some examples only with pro-adipogenic factors, unidirectional block was observed (**bottom right**).

**Figure 6:**
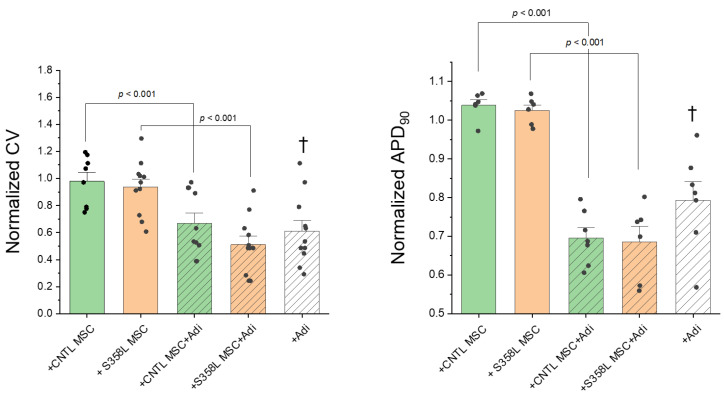
Summary data for conduction velocity (CV, **left**) and APD_90_ (**right**) when S358L cardiac myocytes were co-cultured under early adipogenic conditions. All CV and APD_90_ values are normalized to values measured in cardiac myocytes alone. Co-culturing with either CNTL (+CNTL MSC, *n* = 8, *n* = 6) or S358L (+S358L MSC, *n* = 11, *n* = 6) MSCs had no effect on CV or APD_90_. Only when pro-adipogenic factors were combined with either CNTL (+CNTL MSC +ADI, *n* = 10, *n* = 7) or S358L (+S358L MSC +ADI, *n* = 11, *n* = 6) MSCs was CV and APD_90_ significantly reduced compared to the absence of pro-adipogenic factors. Finally, S358L cardiac myocytes cultured with pro-adipogenic factors alone (+Adi, *n* = 11, *n* = 7) demonstrated a similar reduction in CV and APD_90_. (†, *p* < 0.02 vs. +S358L MSC and +CNTL MSC).

Given that the S358L mutation in myocytes did not demonstrate any obvious abnormal electrophysiological phenotype, we assessed the role of early adipogenic conditions that includes MSCs (fibro-fatty precursor cells) and pro-adipogenic factors. S358L myocyte monolayers were co-cultured with S358L MSCs on top, 4 days before recordings were made. Shown in Figure 5 are representative examples of action potential traces and activation maps. The addition of S358L MSCs had little to no effect on APD and CV (top right) compared to S358L myocytes alone (top left). Interestingly, only when adipogenic conditions were added was an abnormal EP phenotype observed, as evidenced by APD shortening and CV slowing (bottom left). Moreover, in some monolayers, unidirectional block was observed when S358L MSCs and adipogenic conditions were added to S358L myocytes (bottom right), as evidenced by excessive isochrone crowding (~4 o’clock position). Summary data (Figure 6) show that there was no significant difference in CV or APD when either CNTL MSCs (green bars) or S358L MSCs (orange bars) were co-cultured with S358L myocytes. In contrast, when pro-adipogenic factors were added to either CNTL (+CNTL MSC + Adi) or S358L (+S358L MSC + Adi) MSC co-cultures a significant decrease in CV and APD was observed. Finally, when pro-adipogenic factors were added to S358L myocyte monolayers alone (white bar) a similar decrease in CV and APD was demonstrated. Similar results were observed when CNTL CM were used (data not shown). These results demonstrate that pro-adipogenic factors are necessary and sufficient to create arrhythmia substrates in myocyte monolayers. 

**Figure 7:**
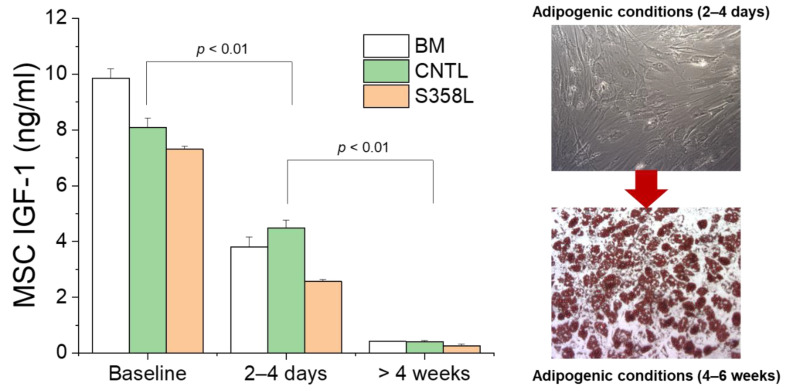
Shown are IGF-1 section levels during adipogenic conditioning of bone marrow (BM) MSCs and MSCs derived from control (CNTL MSC) and S358L (S358L MSC) iPSCs. After 2–4 days of adipogenic conditioning (*n* = 3, *n* = 6, *n* = 6), IGF-1 levels are significantly reduced compared to before adipogenic conditioning was initiated (baseline, *n* = 3, *n* = 3, *n* = 3). After >4 weeks of adipogenic conditioning, when MSCs have differentiated to adipocytes (bottom right, *n* = 5, *n* = 4, *n* = 6), IGF-1 levels are negligible compared to baseline.

We have previously shown that MSCs remodel in disease as evidenced by a decrease in IGF-1 secretion [15]. Given that TMEM43 mutations are associated with enhanced adipogenesis, we determined the effect of pro-adipogenic factors on MSC IGF-1 secretion. Figure 7 shows a progressive and significant decline in the secretion of IGF-1 for BM, CNTL, and S358L MSCs when they were initially exposed to adipogenic conditioning and eventually transitioned to adipocytes by >4 weeks. Importantly, the initial drop in IGF-1 levels secreted by MSCs in the acute initial phase 2–4 days after treatment coincided with no significant structural changes as evidenced by the lack of adipogenesis (Figure 7, top right). By 4 weeks, IGF-1 expression was negligible, and adipocytes were evidenced by fat vacuoles that stain red with Alizaren red O (Figure 7, right bottom). MSCs treated with pro-adipogenic factors typically showed no evidence of adipogenesis until >2 weeks of exposure.

**Figure 8:**
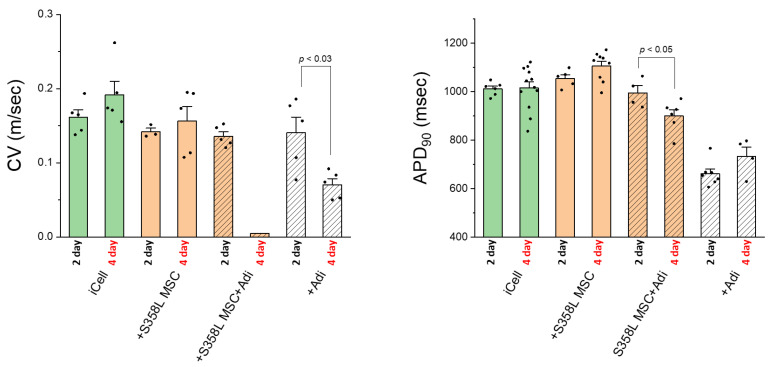
Summary data for conduction velocity (CV, left) and APD_90_ (right) when iCell cardiac myocytes (iCell CM) were co-cultured under 2 or 4 days of early adipogenic conditioning. Co-culturing with S358L (+S358L MSC) MSCs (orange bars) had no effect on CV or APD_90_ at 2 or 4 days. Only when pro-adipogenic factors alone (white hatched bars) or when combined with S358L (+S358L MSC + ADI) MSCs (orange hatched bars) was CV and APD_90_ significantly reduced at 4 days compared to 2 days. For CV: iCell, *n* = 5, *n* = 5; +S358L MSC, *n* = 3, *n* = 5; +S358L MSC + Adi, *n* = 5, *n* = 5; +Adi, *n* = 5, *n* = 5. For APD_90_: iCell, *n* = 6, *n* = 12; +S358L MSC, *n* = 5, *n* = 9; +S358L MSC + Adi, *n* = 4, *n* = 6; +Adi, *n* = 7, *n* = 4.

To better understand the early acute effects of adipogenic conditioning on myocyte monolayers, we studied exposure to pro-adipogenic factors for 2 days vs. 4 days in control iCell myocyte monolayers with and without S358L MSCs. Figure 8 shows that compared to myocytes alone, S358L MSCs (orange bars) had no effect on CV and APD at 2 and 4 days. With adipogenic conditioning added (orange hatched bars), CV was not affected at 2 days, but was not measurable at 4 days due to poor signal quality. Similarly, APD was not affected at 2 days but was significantly decreased by 4 days. Importantly, these changes correspond to the decrease in IGF-1 expression in MSCs exposed to pro-adipogenic factors over a similar time course (Figure 7). These results suggest that the acute decrease in CV and APD observed in adipogenic conditions (+S358L MSC + Adi) may be due to reduced IGF-1 expression in MSCs. Adipogenic conditioning alone (white bars) exhibited a similar but less severe effect, suggesting that a decrease in MSC IGF-1 expression from 2 to 4 days is not fully responsible. This early protection provided by MSCs was independent of whether they had the S358L mutation or not (data not shown).

**Figure 9:**
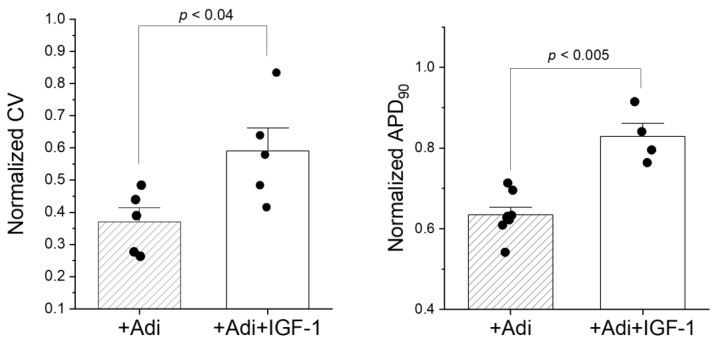
Summary data for conduction velocity (CV, left, *n* = 5, *n* = 5) and APD_90_ (right, *n* = 8, *n* = 4) when cardiac myocytes were co-cultured with pro-adipogenic factors that did (solid white bars) and did not (hatched bars) include IGF-1 supplementation. All CV and APD_90_ values are normalized to values measured in cardiac myocytes alone.

It is possible that pro-adipogenic factors reduced APD and CV in myocytes independent of IGF-1 expression. To test this possibility, myocyte monolayers without MSCs were exposed to pro-adipogenic factors for 4 days and treated with and without IGF-1. Figure 9 shows that treating myocyte monolayers with IGF-1 mitigated APD shortening and CV reduction. This effect by IGF-1 was also observed in the presence of S358L MSCs (not shown). Taken together, these results suggest that MSCs likely attenuate acute arrhythmogenic effects of pro-adipogenic factors initially via IGF-1; however, this protective effect is lost as they remodel and commit to the adipogenic pathway.

## 4. Discussion

Herein, we show that cardiac myocytes derived from patient-specific iPSCs with the ARVC5 TMEM43 p.Ser358Leu (S358L) mutation alone may not be sufficient to replicate the clinical phenotype. In contrast, fibro-fatty conditions including, mainly, pro-adipogenic factors can create arrhythmogenic substrates within just a few days; long before structural abnormalities arise. Taken together, these findings suggest that early adipogenic conditions may be a mechanism of early, concealed arrhythmogenesis associated with ARVC.

### 4.1. Early Arrhythmia Substrates Are Independent of the S358L Mutation in Myocytes

Clinical studies in TMEM43 patients demonstrate a very severe arrhythmia phenotype [7]. Importantly, an early, concealed phase of enhanced arrhythmogenesis in the absence of significant structural abnormalities is a recognized clinical presentation for ARVC patients [2]. We observed significant arrhythmia substrates, including slow impulse conduction, unidirectional block, and APD shortening, which can be caused by ion channel dysfunction [24] as seen in other disease associated with arrhythmia [25,26]. Interestingly, these substrates were observed <4 days after myocyte monolayers were exposed to adipogenic conditions in the absence of structural abnormalities. This does not exclude the possibility of cell-to-cell uncoupling, which could explain the changes in CV we observed. Similar arrhythmia substrates, including non-uniform conduction and fractionated electrograms, have been observed during the early concealed arrhythmia phase in ARVC patients [27]. Importantly, however, we did not observe any arrhythmia substrates in TMEM43 S358L monolayers in the absence of adipogenic conditions over a similar duration, suggesting the mutation alone in myocytes is not sufficient to explain early arrhythmogenesis. This is not an uncommon finding, as recently reported in TMEM43 S358L mice and iPSC derived cardiac myocyte models that either require structural abnormalities or a pharmacological stimulus, respectively, before any electrophysiological phenotype is observed [8]. The same can be said for other ARVC mutations, where no significant phenotype was observed until environmental factors were introduced such as exercising PKP2 mice [28] or exposing patient-specific iPSC-derived myocytes to adult-like metabolism [11]. However, this is not a universal observation, as Siragam et al. [29] showed that overexpression of the TMEM43 S358L mutation in HL-1 cells exhibited reduced CV in monolayers, and other ARVC models exhibit severe clinical phenotypes in the absence of any triggers [30,31]. These reports may be different from ours due to the disease model used (e.g., KO or not) or the expression system (HL-1 or iPSC-derived myocytes), or merely a reflection of the variable expressivity associated with ARVC mutations. Nonetheless, our results suggest that pro-adipogenic conditions alone can create important arrhythmia substrates early, before any significant structural changes. Finally, it is possible that other arrhythmia substrates we did not test for (e.g., calcium dysregulation) exist [32].

### 4.2. Adipogenic Conditions and Arrhythmia Substrates

We found that adipogenic conditions, including PPAR agonists, are an important cause of abnormal impulse conduction and APD shortening before structural changes, which to the best of our knowledge has not been previously reported. The TMEM43 S358L mutation can interfere with AKT signaling and activates GSK3β, which inhibits β-catenin–dependent transcription [8,33]. As a result, canonical Wnt/GSK3β/β-catenin signaling is suppressed and adipogenic, including PPAR signaling, is enhanced [9]. Accordingly, disturbances in PPAR signaling have been associated with ARVC [10]. Furthermore, exercise and inflammation are strongly associated with ARVC (including TMEM43 [5]) and can involve aberrant GSK3β signaling [34]. Finally, pro-adipogenic factors have been used previously to demonstrate calcium dysregulation and lipogenesis in myocytes derived from iPSCs with a PKP2 mutation [11], but important arrhythmia substrates such as reduced CV and APD were not reported. It is unlikely that the PPAR activators we used had a direct and acute effect on ion channels that could explain the decrease in CV and APD we observed, primarily because measurements were made in the absence of pro-adipogenic factors. Additionally, reported direct effects of PPAR activation on I_Na_ [35], connexin43 [36], and repolarizing currents [37] cannot explain the slow CV and APD shortening we observed. Therefore, it is likely that pro-adipogenic factors act upstream and have a significant effect on CV and APD in the absence of any significant structural remodeling

Several studies suggest that MSCs are a precursor for ARVC fibro-fatty infiltrate, and they are also very sensitive to Wnt signaling [18,38], so any disruption could enhance adipogenic signaling in MSCs prior to adipocyte differentiation. Similarly, fibro-adipocyte progenitor cells have been shown to have enhanced adipogenesis upon deletion of the desmosome protein desmoplakin [13]. Given that resident cardiac MSCs exist in the normal heart [15,16] and that TMEM43 is highly expressed in MSCs (Figure 2), suggest they may be an important source of adipogenic conditions. However, we did not notice any worsening of arrhythmia substrates when MSCs, with or without the S358L mutation, were co-cultured with myocytes <4 days. This raises the question of whether MSCs contribute to arrhythmogenesis in the early concealed arrhythmia phase before structural remodeling (e.g., adipocyte differentiation). It is possible that reduced secretion of IGF-1 in MSCs within the first few days of enhanced adipogenic signaling (Figure 7), is causally related to the arrhythmia substrates we observed. 

### 4.3. The Role of IGF-1 in Early Arrhythmogenesis in ARVC

In the present study, we show that IGF-1 mitigated CV slowing and APD shortening when myocytes were exposed to pro-adipogenic factors. In contrast, however, we have previously shown that IGF-1 mitigated APD prolongation when myocytes were exposed to failing MSCs [15]. This opposite effect on APD suggests that the mechanism is unrelated to a direct action on ion channels. Rather, it suggests that IGF-1 acts upstream to protect against disease-associated pathophysiology. Crucially, this raises the possibility that during enhanced adipogenic signaling in ARVC, IGF-1 expression in cardiac MSCs is rapidly decreased, and as a result some protection against arrhythmia is lost. IGF-1 expression in myocytes may also decrease; however, we have previously shown that this might be lower compared to MSCs [15]. This early loss of protection may explain, in part, the early concealed arrhythmia phase in ARVC in the presence of enhanced exogenous adipogenic signaling, for example, by initial myocyte necrosis and inflammation [39]. Indeed, some patients with ARVC exhibit symptoms of myocarditis in the early stages, which can be inflammatory [40] or autoimmune mediated [41]. For other disease, such as myocardial infarction, bone marrow-derived mononuclear cells used for treatment release IGF-1, which blocks cardiomyocyte apoptosis [42]. Similarly, overexpression of IGF-1 in MSCs has been shown to improve their therapeutic benefit [43]. Finally, pro-adipogenic factors, such as PPAR activators can suppress IGF-1 activity [44], which may explain why adding IGF-1 in the presence of pro-adipogenic factors was able to partly reverse the effects we observed. Additional studies are required to determine the mechanistic role of IGF-1 in early arrhythmogenesis associated with ARVC.

## 5. Limitations

We did not perform our experiments in freely (i.e., non-attached) contracting myocytes. It is possible that iPSC-derived cardiac myocytes cultured as more mature engineered tissue constructs might enhance ARVC phenotypes [45]. Nevertheless, we would still expect adipogenic conditions to play a significant role. Although we show that levels of IGF-1 change as MSCs are exposed to adipogenic conditions and IGF-1 can regulate arrhythmia substrates, we cannot rule out that other growth factors and cytokines are playing a role. Finally, the duration of co-culturing was limited to no more than 4 days because MSC proliferation can significantly overwhelm myocytes.

## 6. Clinical Implications

Our results provide a possible explanation for the early concealed arrhythmia phase associated with ARVC, when sudden cardiac arrest/death due to ventricular arrhythmia occurs before overt structural abnormalities are evident. We show that an adipogenic shift in metabolism associated with TMEM43 and, possibly, other ARVC mutations is critical to the pathogenesis of the clinical phenotype, both electrophysiological and structural. It is conceivable, therefore, that metabolic dysfunction and inflammatory status may worsen the clinical phenotype [46,47]. Accordingly, diet modification and targeting inflammatory cytokines may mitigate progression. Our results also suggest that maintaining IGF-1 levels early in the progression of ARVC may be beneficial in preventing arrhythmia before structural remodeling occurs. This work should prompt additional basic, translational, and clinical studies evaluating early adipogenic signaling, IGF-1, and related metabolic dysregulation in patients with ARVC mutations. A better understanding of these mechanisms could lead to more personalized assessment of metabolic status and treatments to prevent arrhythmia in patients with ARVC.

## Data Availability

The data presented in this study are available from the corresponding author by reasonable request.
